# A cystic shaped metastatic osteosarcoma in heart: an unusual view

**DOI:** 10.1093/ehjcr/ytad545

**Published:** 2023-11-07

**Authors:** Mustafa Ucar, Ali Yasar Kilinc

**Affiliations:** Cardiology Department, Celal Bayar University, Uncubozköy District, 5526 Street, No:8/4, Yunusemre, Manisa 45030, Turkey; Cardiology Department, Arnavutkoy State Hospital, Merkez District, No:1165, Arnavutkoy, Istanbul 34275, Turkey

## Case description

**Figure 1:**
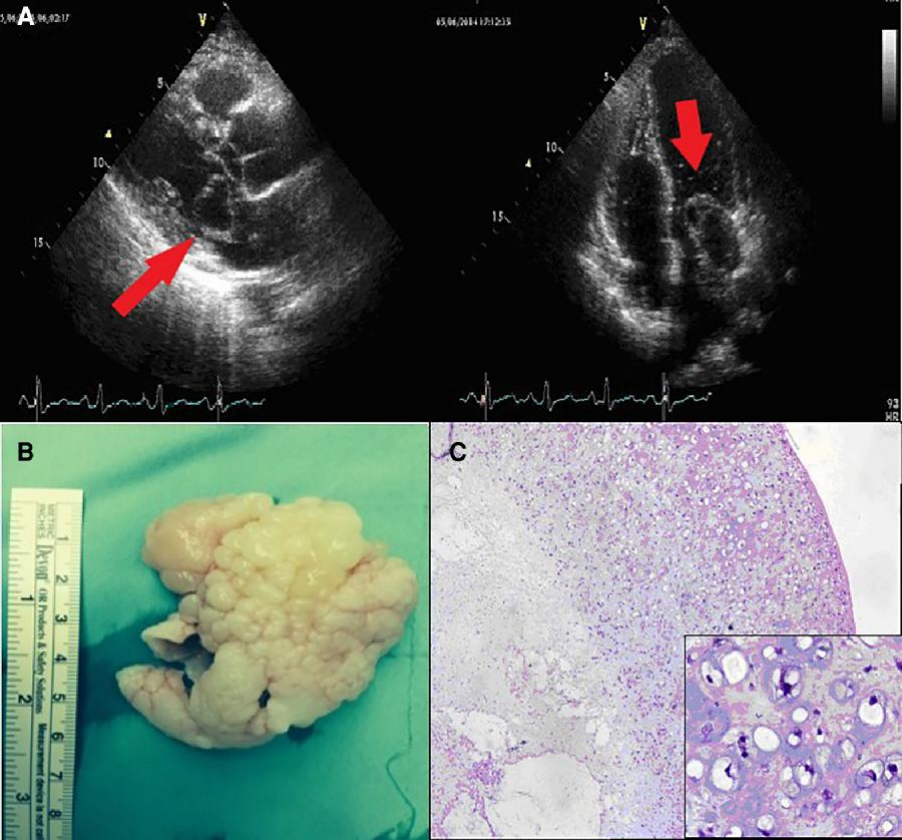
(*A*) Cystic mass was originating from the left superior pulmonary vein and prolapsing to the left ventricle (red arrow). (*B*) Grossly, cauliflower-shaped mass with myxoid appearance was seen. (*C*) Tumour was composed of hyperchromatic cells within the chondroid matrix which formed lobules. The centre of the tumour had necrosis. Tumor cells lie in the lacunae and have severe atypia (inset).

A 32-year-old man was admitted to the hospital complaining of palpitation and dsypnea for 2 weeks. He had a past history of mandibular tumour 8 years ago which was diagnosed as ‘Osteosarcoma, chondroblastic type’ and received chemotherapy after surgical resection. On admission his vital signs were normal. He had a diastolic murmur at the left sternal border. Transthoracic echocardiography revealed a multilocular cystic mass originating from the left superior pulmonary vein in left atrium that prolapsing left ventricle at diastolic period (*Figure 1A*). There was a 19 mmHg peak gradient and a 10 mmHg mean gradient across the mitral valve. The patient underwent a minimal invasive operation for the excision of mass. On gross examination, 6 × 4.5 × 3 cm sized cauliflower-shaped creamy mass with myxoid cut surface was defined (*Figure 1B*). Microscopically, tumour was composed of spindle and round tumour cells that lie in the lacunae and form lobules within an abundant extracellular chondroid matrix. The centre of the tumour had necrosis and towards the periphery tumour became hypercellular. Severe cytological atypia and mitotic figures were observed. By these findings, the patient was diagnosed as ‘Malignant mesenchymal tumour with chondroid differentiation’ and considered as ‘Cardiac metastasis of osteosarcoma, chondroblastic type’ (*Figure 1C*).

Most frequent malignant cardiac tumours are sarcomas.^1^ Metastatic sarcomas to the heart are more likely to involve the myocardium. In only 5% of cases, endocardium or chamber cavities are involved, as in our patient. All types of sarcomas are known to metastasize and evidently, this occurs via the haematogenous route.^2^ Generally, metastatic tumour is a relative contraindication for surgery. In this case, we preferred to perform surgery, because the mass was mimicking mitral stenosis by prolapsing through the mitral valve during diastole and a mass in the cardiac cavity can embolize. According to our knowledge, this case seems to be the first cystic-shaped osteosarcoma metastasis on transthoracic echocardiography.

**Consent:** The authors confirm that written consent for submission and publication of this case report including images and associated text has been obtained from the patient in line with COPE guidance.

**Funding:** None declared.

## Data Availability

The data underlying this article will be shared on reasonable request to the corresponding author.

## References

[1] Sutsch G, Jenni R, von Segesser L, Schneider J. Heart tumors: incidence, distribution, diagnosis. Exemplified by 20,305 echocardiographies. Schweiz Med Wochenschr 1991;121:621–629. 2047823

[2] Silver MD, Gotlieb AI, Schoen FJ. Cardiovascular Pathology. 3rd ed. New York: Churchill Livingstone Company; 2001. p600–601.

